# Underweight, overweight, and tobacco use among adolescents aged 12–15 years: Evidence from 23 low-income and middle-income countries

**DOI:** 10.18332/tid/133932

**Published:** 2021-05-12

**Authors:** Qian Wang

**Affiliations:** 1 School of Public Health, Shanghai Jiao Tong University School of Medicine, Shanghai, China

**Keywords:** underweight, overweight, tobacco use, adolescents, low- and middle-income countries

## Abstract

**INTRODUCTION:**

Compared with the number of studies in adults, body weight in relation to tobacco use has been understudied in the adolescent population. This study aimed to examine the association between underweight, overweight and tobacco use in low- and middle-income countries.

**METHODS:**

Data were derived from the Global School-Based Student Health Survey (GSHS). Data from 71176 adolescents aged 12–15 years residing in 23 countries were analyzed. The Centers for Disease Control and Prevention (CDC) 2000 growth charts were used to identify underweight, normal weight, and overweight/ obesity. Weighted age- and gender-adjusted prevalence of weight categories and tobacco use was calculated. Multivariate logistic regression analysis was performed to estimate the association between weight categories and tobacco use for each country, controlling for covariates. Pooled odds ratios and confidence intervals were computed using random- or fixed-effects meta-analyses.

**RESULTS:**

A significant association between weight categories and tobacco use was evident in only a few countries. Adolescents reporting tobacco use in French Polynesia, Suriname, and Indonesia, had 72% (95% CI: 0.15–0.56), 55% (95% CI: 0.24–0.84), and 24% (95% CI: 0.61–0.94) lower odds of being underweight, respectively. Adolescents reporting tobacco use in Uganda, Algeria, and Namibia, had 2.30 (95% CI: 1.04–5.09), 1.71 (95% CI: 1.25–2.34), and 1.45 (95% CI: 1.00–2.12) times greater odds of being overweight/obese, but those in Indonesia and Malaysia had 33% (95% CI: 0.50–0.91) and 16% (95% CI: 0.73–0.98) lower odds of being overweight/obese.

**CONCLUSIONS:**

The association between tobacco use and BMI categories is likely to be different among adolescents versus adults. Associating tobacco use with being thin may be more myth than fact and should be emphasized in tobacco prevention programs targeting adolescents.

## INTRODUCTION

Tobacco use has long been recognized as a leading risk factor for non-communicable diseases^1^. Since the World Health Organization’s Framework Convention on Tobacco Control (FCTC) came into effect in 2005, many high-income countries (HICs) legislated the majority of WHO policies for tobacco control, and smoking prevalence continued to decline in these countries^2,3^. Although middle-income countries also increased the number of legislated tobacco control policies during this time, yet, upper middle-income countries are now estimated to have the highest smoking prevalence (24%)^2,3^. Low-income countries lagged behind both the high-income and middle-income countries in the speed and scope of tobacco-control policy legislation^3^, and over a third of low-income countries had insufficient national data to consistently document trends in smoking prevalence^2^. Moreover, compared with high-income countries, low- and middle-income countries are increasingly burdened by tobacco use related mortalities^4^. As a result, tobacco use is increasingly becoming a looming public health challenge threatening low- and middle-income countries.

The association between body weight and tobacco use, especially cigarette smoking in the adult population has been the focus of numerous studies. Nicotine, the chief addictive substance in tobacco products, can contribute to weight loss by reducing appetite and increasing energy expenditure^5^. In the adult population, cross-sectional and longitudinal studies appear to support an inverse relationship between cigarette smoking and body weight, as prolonged consumption of nicotine was associated with increased central adiposity^6^. Compared with adulthood, adolescence is characterized by experimental use of all forms of tobacco products, and concerns about being overweight or obese are often cited as a reason for smoking initiation among adolescents^6,7^. Some studies have proposed that young people may perceive smoking as a weight control method; overweight/obese youth may perceive smoking as a method of self-medication to relieve weight-associated psychological distress; and smoking can also be perceived as a means to build social relationships with others, especially by overweight/ obese adolescents experiencing weight-related social isolation^8^. However, contrary to the popular belief, there is insufficient evidence suggesting that smoking is associated with significant weight loss among adolescents and young adults^9,10^, perhaps because the weight-reducing effect of nicotine consumption is accumulated over time, only becoming significantly evident in adulthood^10^.

Studies supporting these findings were mainly conducted among adolescents in western countries, the association between body weight and cigarette smoking among adolescents in low- and middle-income countries has been underexplored. Therefore, this study aimed to draw attention to this issue by utilizing the Global School-based Student Health Survey (GSHS), which collects data from adolescents in a large number of low- and middle-income countries. The GSHS is one of the very few surveys that collect information on health-related behaviors of adolescents residing in countries where national surveillance systems are not yet established. It was argued that patterns of tobacco use should be assessed within the unique social and cultural contexts^11^. Unlike in western countries where cigarette smoking is the predominant form of tobacco use, use of non-cigarette tobacco products is the culturally predominant form of tobacco consumption in many low- and middle-income countries^12^. Thus, the key independent variable in this study was tobacco use, including cigarettes, as well as other nicotine-containing tobacco products.

Body weight can be represented by the body mass index (BMI, kg/m^2^). BMI has become the most practical, inexpensive, and non-invasive anthropometric measure^13^, especially in population-based surveys where direct measures of body weight and height were not feasible. It was found to correlate well with objectively measured body weight and height, body fat and cardiovascular risk factors in children and adolescents^14-16^. BMI values for children and adolescents aged <19 years are usually adjusted for age and gender, and expressed as a percentile showing the relative position of a child’s or an adolescent’s weight and height among peers of the same gender and age. For the adolescent population, two of the most commonly referenced BMI-for-age growth charts are the 2000 Centers for Disease Control and Prevention (CDC) growth charts and the 2007 growth charts (5–19 years) from the World Health Organization (WHO)^17,18^. Both charts were created based on the 1977 growth charts developed by the US National Center for Health Statistics (NCHS). However, the reference population as well as the statistical methods used to construct the two growth charts differed. The 2000 CDC growth charts were constructed based on updated reference population used to create the 1977 NCHS growth charts^17^, whereas the 2007 WHO growth charts (5–19 years) were constructed based on merging the same reference population used in creating the 1977 NCHS growth charts with the 2006 WHO Child Growth Standards for under-fives^18^. In regard to statistical methods, the z-scores in the 1977 NCHS growth charts were based on a fixed standard deviation value above and below the median, whereas the 2000 CDC growth charts were based on the LMS (lambda-mu-sigma) smoothing method allowing for a changing standard deviation^17^. For children aged <5 years, the 2006 WHO Child Growth Standards were recommended for use^19^; there is also evidence supporting using the 2006 WHO Child Growth Standards over the 2000 CDC growth charts in developing countries^20,21^. However, for youth aged 5–19 years, despite several studies found that the 2007 WHO growth chart (5-19) overestimated obesity prevalence or underestimated stunting prevalence^22-24^, there is almost no evidence endorsing the use of one growth chart over the other. Because the 2000 CDC growth charts were based on more contemporary data and statistical procedures, they were used to derive BMI-for-age percentiles for adolescents aged 12–15 years in the current study. Findings of this study may enhance our collective understanding of the body-weight–tobacco-use link among adolescents worldwide.

## METHODS

### Sample

Data were derived from the Global School-based Student Health Survey (GSHS). The GSHS was developed by the World Health Organization in conjunction with United Nations-affiliated organizations, as well as the United States Centers for Disease Control and Prevention (CDC). It primarily surveys adolescents aged 13–17 years residing in low-or middle-income countries. In each participating country, ethical approval was obtained from the Ministry of Education or a relevant institution ethics review committee. A two-stage cluster sampling design was utilized to obtain nationally representative samples, where schools were first selected with probability proportional to enrollment size, classes within selected schools were randomly chosen during the second stage, and all students in the selected classes were eligible to participate. Verbal or written consents were obtained from participants and their guardians before beginning of the survey. More details about the GSHS can be found elsewhere (https://www.cdc.gov/gshs/pdf/GSHSOVerview. pdf). The target population of the present study was adolescents aged 12–15 years.

### Measures

#### Body mass index (BMI) categories – outcome variable

Prior to survey administration, survey staff typically measured participants’ weight and height and wrote the numbers on paper for students to enter during survey administration^25^. BMI was calculated as weight (kg) divided by height squared (m^2^) (kg/m^2^). The 2000 CDC growth charts were used to derive age-and gender-adjusted BMI percentiles^17^. According to the growth charts, underweight was defined as a BMI score <5th percentile, normal weight was defined as a BMI score ≥5th percentile to <85th percentile, overweight was defined as a BMI score ≥85th percentile, and obese were defined as a BMI score ≥95th percentile^17^. Participants in the normal weight category were used as the reference group in the multivariate analyses. Because the percentage of adolescents who were obese was low, those who were obese were not categorized separately from those who were overweight. BMI was analyzed as a categorical variable in the current study to help identify those with abnormal weight status and opportunities for interventions.

#### Tobacco use – exposure variable

Tobacco use was assessed by the combined responses to two questions: ‘During the past 30 days, on how many days did you smoke cigarettes?’ and ‘During the past 30 days, on how many days did you use any tobacco products other than cigarettes, such as a pipe, rolled tobacco leaves, snuff, or chewing tobacco?’. Response options included: 0, 1–2, 3–5, 6–9, 10–19, 20–29, and 30 days. Because the majority of participating adolescents reported non-use during the past 30 days, thus skewing the distribution of the variable, responses were dichotomized to distinguish those (tobacco users) reporting at least 1 or 2 days to either question from those reporting non-use to both questions. This was done consistently with other studies^26,27^, moreover, the dichotomized variable was also embedded in the original GSHS datasets as ‘Percentage of students who currently used any tobacco product (on at least 1 day during the 30 days before the survey)’.

### Covariates

Variables commonly associated with BMI were included as covariates: age (12, 13, 14, and 15 years), gender (male vs female), food insecurity (used as a proxy measure of socioeconomic status) were assessed by the frequency of going hungry ‘because there was not enough food in your home’ (‘most of the time’ or ‘always’ vs ‘never’, ‘rarely’, ‘sometimes’), fruit and vegetable consumption (≥5 times per day vs <5 times per day) was assessed by summing the number of times of having fruits or vegetables per day during the past 30 days (used as a proxy for adequate daily intake recommended by the WHO)^28^, physical activity was assessed by the number of days physically active for at least 60 min per day during the past week (≥5 days/week vs <5 days/week).

### Data analysis

Exclusion criteria were: 1) countries that did not include all variables used in this analysis were excluded; and 2) countries that had more than 14% of data missing on all variables used in the analysis^29,30^. For final analysis, 23 countries were included and grouped by World Bank income level in the corresponding survey year into: low-income countries (n=3), lower middle-income countries (n=9), upper middle-income countries (n=6), and high-income countries (n=5). Only the latest survey was used if a country conducted the GSHS twice or more.

Survey analysis procedures (SAS version 9.4, SAS Institute, Cary, NC) were utilized to account for the complex sampling design of the GSHS. According to the CDC growth charts, age- and gender-adjusted mean BMI percentiles were calculated for each country, and BMI categories were derived from calculated BMI percentiles. Age- and gender-adjusted percentages were also calculated for all other categorical variables. Within each country, the association between BMI categories and tobacco use was estimated via multivariate logistic regression. The pooled total and regional association between BMI categories and tobacco use was estimated via meta-analysis in Stata version 15.1 (StataCorp LLC, Texas, USA), with Higgins’ I^2^ as the indicator of between-country heterogeneity. Between-country heterogeneity is typically considered low when Higgins’ I^2^ is 25%–50%^31^. Because there was significant heterogeneity between countries, a random-effects model was constructed to obtain the pooled estimate of the association. The level of statistical significance was set at p<0.05.

## RESULTS

### Sample characteristics

The sample consisted of 71176 adolescents aged 12– 15 years residing in 23 countries (Table 1). Eighteen countries (78.3%) were in the low- to middle-income categories, and 5 countries (21.7%) were in the high-income category (Table 1). Age- and gender-adjusted mean BMI percentiles ranged from 36.2% (SE=1.9) in Namibia to 80.1% (SE=0.6) in Tonga (Table 2). Prevalence of underweight (BMI <5th percentile) ranged from 0.6% (95% CI: 0.3–0.8) in Tonga to 17.9% (95% CI: 14.4–21.4) in Namibia (Table 2). Prevalence of overweight (BMI ≥85th percentile) ranged from 6.1% (95% CI: 4.8–7.4) in Pakistan to 57.8% (95% CI: 55.2–60.4) in Tonga (Table 2). Age- and gender-adjusted prevalence of tobacco use also varied across countries, ranging from 1.1% (95% CI: 0.3–1.9) in Benin to 14.8% (95% CI: 11.7–17.9) in Syria (Table 2).

**Table 1:** Survey characteristics by country, Global School-based Student Health Survey 2003–2017 (N=71176)

*Income level ^a^*	*Country*	*Survey year*	*World region*	*Response rate (%) ^b^*	*Sample size (n) ^c^*
LIC	Benin	2016	AFR	78	664
	Uganda	2003	AFR	69	1697
	Syria	2011	EMR	97	2715
LMIC	Algeria	2011	AFR	98	3275
	China (Beijing)	2003	ASIA	99	1966
	Pakistan	2009	ASIA	76	4624
	Indonesia	2015	ASIA	94	7941
	Mongolia	2013	ASIA	88	3497
	Thailand	2015	ASIA	89	3656
	Bolivia	2012	AME	88	2562
	Guyana	2009	AME	76	1775
	Honduras	2012	AME	79	1350
UMIC	Namibia	2013	AFR	89	1693
	Iraq	2012	ASIA	88	1370
	Malaysia	2012	ASIA	89	15419
	Tonga	2017	ASIA	90	1848
	Jamaica	2017	AME	60	927
	Suriname	2016	AME	83	1374
HIC	Bahrain	2016	ASIA	89	5085
	Brunei	2014	ASIA	65	1726
	French Polynesia	2015	AME	70	1726
	Mauritius	2017	AFR	84	1790
	Uruguay	2012	AME	77	2496

LIC: low-income countries. LMIC: lower middle-income countries. UMIC: upper middle-income countries. HIC: high income countries. AFR: African region. AMR: Region of the Americas. EMR: Eastern Mediterranean Region.

a Country income level was based on the World Bank classification at the year of the survey in the respective countries.

b Response rate: school response rate × student response rate.

c Students aged 12–15 years.

**Table 2:** Age- and gender-adjusted prevalence of BMI categories and tobacco use among adolescents aged 12–15 years in 23 countries, Global School-Based Student Health Survey 2003–2017 (N=71176)

*Income level ^a^*	*Country*	*BMI ^b,c^*				*Tobacco use ^c,d^*
		*Mean percentile (S.E.)*	*<5th percentile*	*5th to <85th percentile*	*≥85th percentile*	
			*% (95% CI)*	*% (95% CI)*	*% (95% CI)*	*% (95% CI)*
LIC	Benin	46.7 (2.6)	10.6 (7.5–13.8)	74.3 (68.8–79.7)	15.1 (9.3–20.9)	1.1 (0.3–1.9)
	Uganda	42.1 (2.5)	12.1 (7.6–16.7)	80.9 (76.4–85.3)	7.0 (4.0–10.0)	1.7 (0.2–3.2)
	Syria	58.0 (1.2)	5.2 (4.1–6.4)	72.8 (70.3–75.2)	22.0 (19.0–25.0)	14.8 (11.7–17.9)
LMIC	Algeria	46.1 (1.1)	8.5 (7.3–9.7)	77.4 (75.5–79.4)	14.1 (12.0–16.1)	4.7 (3.6–5.9)
	China (Beijing)	51.8 (1.5)	6.8 (5.6–8.0)	72.1 (68.0–76.3)	21.1 (17.0–25.2)	4.0 (2.4–5.6)
	Pakistan	36.7 (1.2)	14.2 (11.6–16.8)	79.7 (77.4–82.0)	6.1 (4.8–7.4)	7.5 (5.2–9.7)
	Indonesia	47.0 (0.9)	10.9 (9.8–12.1)	71.3 (69.5–73.2)	17.7 (15.7–19.8)	7.3 (5.5–9.0)
	Mongolia	48.7 (0.8)	4.2 (3.5–5.0)	83.9 (82.4–85.4)	11.9 (10.2–13.6)	3.3 (2.5–4.2)
	Thailand	51.3 (1.0)	9.3 (8.1–10.6)	67.2 (64.5–69.9)	23.5 (20.6–26.3)	7.4 (5.3–9.4)
	Bolivia	61.3 (0.9)	1.7 (1.2–2.1)	75.3 (72.6–78.0)	23.0 (20.2–25.8)	7.2 (4.9–9.5)
	Guyana	49.1 (1.6)	9.6 (6.9–12.3)	72.5 (68.5–76.5)	17.9 (14.6–21.2)	11.3 (2.6–19.9)
	Honduras	54.5 (1.2)	4.1 (2.8–5.3)	74.6 (70.6–78.5)	21.4 (17.4–25.4)	8.7 (5.6–11.8)
UMIC	Namibia	36.2 (1.9)	17.9 (14.4–21.4)	73.6 (70.5–76.7)	8.5 (5.1–12.0)	2.2 (1.2–3.2)
	Iraq	57.9 (1.4)	5.8 (4.3–7.4)	66.4 (63.3–69.6)	27.8 (24.0–31.5)	4.7 (3.2–6.2)
	Malaysia	54.6 (0.3)	9.8 (9.3–10.2)	60.6 (59.5–61.7)	29.7 (28.7–30.6)	3.1 (2.5–3.8)
	Tonga	80.1 (0.6)	0.6 (0.3–0.8)	41.7 (39.1–44.2)	57.8 (55.2–60.4)	9.1 (6.9–11.3)
	Jamaica	59.9 (1.2)	4.0 (2.7–5.4)	67.9 (64.5–71.4)	28.0 (24.9–31.1)	9.5 (2.6–16.3)
	Suriname	59.5 (0.6)	7.5 (6.3–8.7)	61.1 (57.9–64.4)	31.1 (28.5–34.1)	3.9 (2.3–5.4)
HIC	Bahrain	68.8 (0.6)	4.6 (3.9–5.4)	49.9 (48.5–51.4)	45.4 (43.8–47.0)	6.4 (5.1–7.7)
	Brunei	68.0 (0.9)	3.3 (2.6–4.0)	53.7 (50.6–56.9)	43.0 (39.7–46.2)	3.5 (2.5–4.6)
	French Polynesia	73.6 (1.4)	1.2 (0.3–2.1)	50.9 (46.8–55.0)	47.9 (43.5–52.4)	8.6 (6.3–10.8)
	Mauritius	54.4 (1.1)	13.8 (12.2–15.4)	55.0 (51.5–58.5)	31.2 (28.0–34.5)	4.8 (3.2–6.3)
	Uruguay	62.8 (0.7)	2.3 (1.7–2.8)	69.3 (66.4–72.2)	28.4 (25.6–31.2)	3.3 (1.7–4.8)

LIC: low-income countries. LMIC: lower middle-income countries. UMIC: upper middle-income countries. HIC: high income countries.

a Country income level was based on the World Bank classification at the year of the survey in the respective countries.

b BMI percentiles were calculated based on the Centers for Disease Control and Prevention 2000 growth charts.

c Estimates were weighted and adjusted for age and gender.

d Past 30-day use of cigarettes and other tobacco products.

Food insecurity was highest in Syria (10.1%; 95% CI: 7.7–12.5) and lowest in Mongolia (1.0%; 95% CI: 0.5–1.4) (Supplementary file Table S1). More than half of respondents in 19 countries reported consuming fruits and vegetables for ≥5 times per day, with the highest percentage of consumption (90.6%; 95% CI: 89.3–92.0) observed in Beijing, China. Fruit and vegetable consumption was lowest among adolescents aged 12–15 years in Benin (19.9%; 95% CI: 18.6–21.1) (Supplementary Table S1). More than 40% of respondents in Uruguay were physically active for at least 60 minutes for ≥5 days within the past week; in comparison, only 8.0% of respondents did the same in Namibia (Supplementary Table S1).

### BMI categories and tobacco use

**Figure 1:**
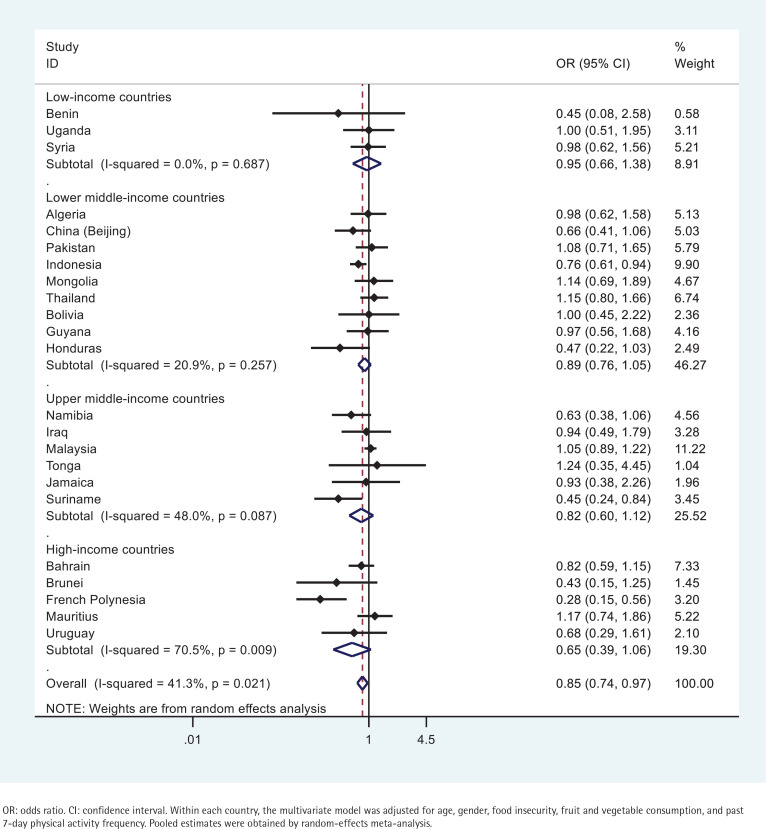
Country-wise association between past 30-day any tobacco use and being underweight estimated by multivariate logistic regression

The association between underweight and tobacco use did not reach statistical significance in The majority of the countries examined (Figure 1). A negative association was evident in only 3 countries: French Polynesia, Suriname, and Indonesia (Figure 1). Respondents engaging in tobacco use in French Polynesia were 72% (95% CI: 0.15–0.56) less likely to be underweight (Figure 1). Respondents in Suriname that engaged in tobacco use were 55% (95% CI: 0.24–0.84) less likely to be underweight (Figure 1). And those engaging in tobacco use in Indonesia were 24% (95% CI: 0.61–0.94) less likely to be underweight (Figure 1).

**Figure 2:**
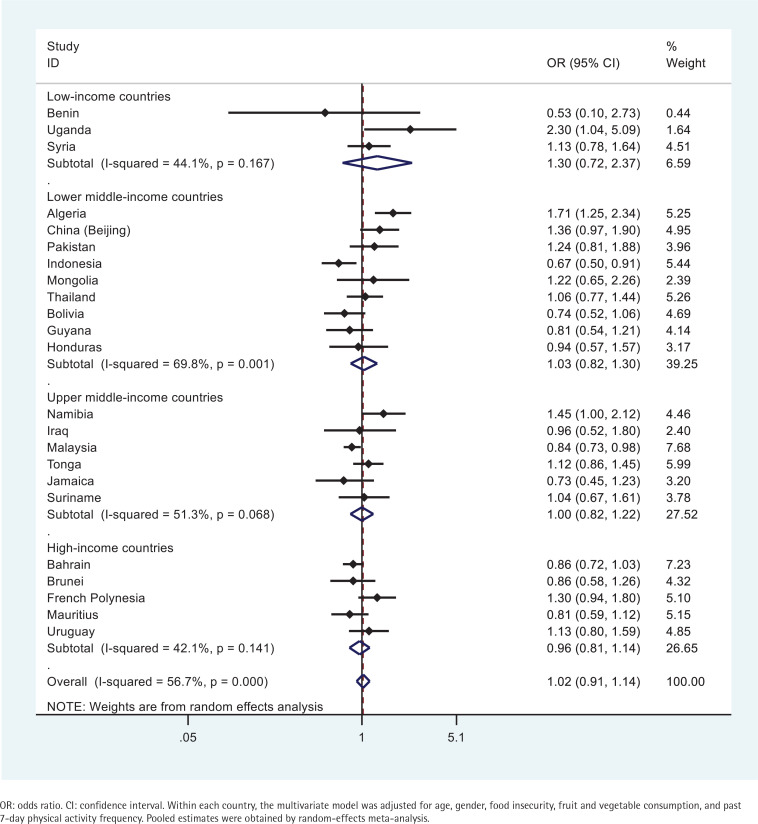
Country-wise association between past 30-day any tobacco use and being overweight/obese estimated by multivariate logistic regression

Similarly, the association between overweight/obesity and tobacco use did not reach statistical significance in the majority of the countries examined (Figure 2). A positive association was observed in Uganda, Algeria, and Namibia (Figure 2). In contrast, a negative association was observed in Indonesia and Malaysia (Figure 2). In Uganda, Algeria, and Namibia, respondents engaging in tobacco use had 2.30 (95% CI: 1.04–5.09), 1.71 (95% CI: 1.25–2.34), and 1.45 (95% CI: 1.00–2.12) times greater odds to be overweight/obese, respectively; while respondents in Indonesia and Malaysia were 33% (95% CI: 0.50–0.91) and 16% (95% CI: 0.73–0.98) less likely to be overweight/obese, respectively (Figure 2).

## DISCUSSION

This was the first study to examine the association of BMI categories with tobacco use among nationally representative samples of adolescents aged 12–15 years residing mainly in low-income and middle-income countries. Existing studies on this association were commonly conducted in western industrialized nations where smoking rates have declined but obesity rates have increased. Overall, the findings point to considerable between-country heterogeneities in the prevalence as well as association of BMI categories and tobacco use.

In six countries (Namibia, Pakistan, Mauritius, Uganda, Benin, and Indonesia), more than 10% of adolescents were underweight, and 4 out of the 6 countries were in the low-income or lower middle-income category. Namibia and Pakistan reported the highest prevalence of underweight adolescents (17.9%; 14.2%), but a comparatively low prevalence of overweight/obese adolescents (8.5%; 6.1%). There has been ample evidence documenting underweight among children under the age of five in Namibia, but limited coverage on its adolescent population. Similarly, both stunting and wasting remain heavily prevalent among children under the age of five in Pakistan. In fact, Pakistan has the second highest infant and child mortality rate in South Asia, with undernutrition as a major contributor^32^. The reasons for such conditions in Pakistan are multidimensional, including congenital abnormalities, maternal undernutrition and early cessation of breastfeeding, gender and income inequality, sanitation related to food preparation, and other sociocultural factors^32^. Underweight and stunting has been extensively documented among children and adolescents aged <16 years in Uganda^33-35^. Adolescents aged 12–15 years in Mauritius were found to exhibit the third highest prevalence of underweight, and the sixth highest prevalence of overweight/obesity among all 23 countries. In another study, a high prevalence of both overweight and thinness was also observed among children aged 9–10 years in Mauritius^36^. Similarly, the comparable prevalence of underweight and overweight/obesity observed among adolescents aged 12–15 years in Benin and Indonesia was consistent with the existing literature^37,38^. This pattern of the dual burden of malnutrition seen in children and adolescents may pose unique public health challenges in these countries.

On the other hand, in four countries (Tonga, French Polynesia, Bahrain, and Brunei), more than 40% of responding adolescents aged 12–15 years were overweight/obese, and 3 out of the 4 countries were in the high-income category. Tonga, an upper middle-income country, had more than half (57.8%) of respondents identified as overweight or obese. These four countries also had a comparatively low underweight prevalence (0.6%–4.6%). Both Tonga and French Polynesia belong to an archipelago of island countries scattered across the Pacific Ocean. The WHO estimated that at least ten of the island countries had more than 50% of the population being overweight or obese, and 40% of the population in the region was diagnosed with a non-communicable disease (i.e. cardiovascular disease, diabetes, and hypertension)^39^. A significant shift away from traditional foods and towards imported, calorie-dense foods was considered a major contributor to the obesity epidemic^39^. In Tonga, it was estimated that over 70% of adults were overweight or obese, and 19.0% of the population was diagnosed with type II diabetes in 2012^40^. The prevalence of overweight/ obesity observed among only adolescents aged 12–15 years accentuated the extent of the obesity epidemic in Tonga. Bahrain and Brunei had one of the highest prevalence of overweight/obese adolescents in their respective region^41,42^. In Bahrain, a rapid shift from traditional foods to those rich in fats and calories was a major contributing factor^43^, but no study has comprehensively examined factors contributing to the overweight/obesity epidemic among children and adolescents in Brunei.

Adolescents in Syria (14.8%) had the highest prevalence of tobacco use among all countries examined. Syria’s tobacco epidemic only came to light when data on tobacco use based on convenience samples and then on nationally-representative samples emerged in the early 2000s^44^. Data collected by the Syrian Center for Tobacco Studies (SCTS) revealed that 51.4% adult men and 11.5% adult women smoked cigarettes daily, while 20.2% adult men and 4.8% adult women engaged in waterpipe tobacco use^44^. The lack of comprehensive tobacco-control policies or resources for cessation and counseling is compounded by political turmoil, highlighting the need for expeditious efforts to resolve this public health crisis in Syria. In 2015, 14.8% of children aged 13–15 years engaged in tobacco use in Guyana^45^, an increase from 11.3% (12–15 years) in 2009 found in the present study. Guyana’s National Tobacco Act, celebrated as the most comprehensive tobacco control law in the nation, was enacted in 2017, and the effectiveness of the Act in reducing smoking prevalence is to be assessed in the coming years^45^.

The current study did not find a consistent association between BMI categories and tobacco use among adolescents across 23 countries. In the few countries where the association was evident, underweight status was associated with lower odds of tobacco use, but the association between overweight/ obesity and tobacco use went in both directions. In Uganda, Namibia, and Algeria, adolescent tobacco users had greater odds of being overweight or obese, whereas in Indonesia and Malaysia, adolescent tobacco users had lower odds of being overweight or obese. Adolescent tobacco users in Indonesia, Suriname, and French Polynesia had lower odds of being underweight. The finding in Malaysian adolescents was consistent with that from another study utilizing the GSHS data, yet, no explanation for the association was provided^46^. In Indonesia, adolescent tobacco users had lower odds of being on both ends of the BMI categories. A longitudinal analysis of 9000 men aged 15–55 years in Indonesia revealed that, smoking was related to weight loss and lower BMI, yet, the size of the effect was too small^47^. No comparable study was conducted to examine this association in the adolescent population in Indonesia.

These results were largely consistent with findings from several meta-analysis reviews^10,48,49^. In one meta-analysis review, findings from 55 studies revealed mixed evidence for an association between smoking and body weight among adolescents, with some studies reporting a positive association while other studies reporting no association^10^. The review found smoking was more evidently correlated with some general weight concerns among female adolescents, but no clear gender patterns were found in the association between smoking and body weight^10^. According to some researchers, a positive association between tobacco use and body weight was mainly attributed to the following factors: some overweight adolescents engaged in tobacco use to control their weight; smoking and obesity-related behaviors such as physical inactivity and sedentary behavior tend to cluster^10^. Besides the above factors commonly observed among adolescents in western countries, another important factor of smoking participation or continuation among adolescents from low- and middle-income countries was cigarette affordability^50^. Compared with adults, adolescents especially those from low-income households tend to be more sensitive to increases in tobacco prices^51^. Furthermore, underweight status or overweight/obesity were both positively associated with household wealth in low- and middle-income countries^47,52,53^. As a result, it is possible that in these countries, underweight adolescents might be less financially capable and overweight/obese adolescents might be more financially capable to afford tobacco products. However, such proposed associations were not consistently observed across all countries in the current study, indicating the need for more research on this topic in the target population.

### Limitations

This study has several limitations. First, most of the variables used in this study were derived from self-report data, which could be subject to recall bias. However, the GSHS uses standardized survey items across participating countries since its inception in 2003, many researchers have utilized data from the GSHS and examined validity of its items. Nonetheless, research using both objectively-measured and self-reported data is needed. Second, due to the proportion of missing data or the absence of questions assessing variables used in the current analysis, data from many countries were excluded from this study. Inclusion of data from more countries may facilitate a more complete view of the association between BMI categories and tobacco use. Third, due to the lack of studies on the BMI-categories–tobacco-use association in the adolescent population in the majority of low-and middle-income countries, we can only speculate on the probable mechanisms.

## CONCLUSIONS

This study aimed to draw attention to the prevalence of BMI categories and their association with tobacco use in the adolescent population worldwide. Considerable between-country heterogeneities were found, with a significant association observed in only a few countries. In countries where a significant association was observed, tobacco use was associated with lower odds of being underweight, yet, its association with overweight/obesity went in both directions. Taken together, these findings imply that the association between tobacco use and BMI categories is likely to be different among adolescents versus adults. That is, the inverse association between body weight and tobacco use commonly observed in adults may not apply to adolescents, thus, associating tobacco use with being thin may be more myth than fact, and such messages should be emphasized in tobacco prevention programs targeting adolescents. Nonetheless, a more complete understanding of this association among adolescents may benefit from studies that are prospective in design and clarify stages of smoking and anthropometric measurements.
