# Supplementary material for: Underweight, overweight, and tobacco use among adolescents aged 12–15 years: Evidence from 23 low-income and middle-income countries
Source: Tob Induc Dis. 2021 May 12;19:37. doi: 10.18332/tid/133932 (PMC8114737; doi:10.18332/tid/133932)
Supplement: Supplementary file 1 [file TID-19-37-s1.pdf]

Supplementary Table S1. Country-wise age- and gender-adjusted prevalence of food insecurity, fruit and vegetable consumption, and physical activity (n=71,176), Global School-Based Student Health Survey, 2003-2017.

Income Level[a]	Country	Food insecurity[b] % (95% CI)[e]	Fruit and vegetable consumption[c] % (95%CI)[e]	Physical activity[d] % (95%CI)[e]
LIC	Benin	2.8 (1.1, 4.5)	19.9 (18.6, 21.1)	8.3 (6.7, 9.9)
	Uganda	2.1 (0.2, 4.1)	45.5 (41.7, 49.2)	12.2 (7.9, 16.5)
	Syria	10.1 (7.7, 12.5)	77.7 (74.7, 80.6)	14.2 (11.5, 16.9)
LMIC	Algeria	5.6 (4.4, 6.9)	74.5 (73.5, 75.5)	15.8 (14.1, 17.5)
	China (Beijing)	2.5 (1.5, 3.5)	90.6 (89.3, 92.0)	35.0 (29.8, 40.2)
	Pakistan	3.7 (2.3, 5.0)	88.3 (83.3, 93.2)	19.3 (6.3, 32.3)
	Indonesia	3.3 (2.5, 4.1)	69.3 (67.9, 70.6)	11.0 (9.4, 12.7)
	Mongolia	1.0 (0.5, 1.4)	51.8 (50.0, 53.6)	25.1 (23.2, 26.9)
	Thailand	2.2 (1.4, 2.9)	62.1 (60.7, 63.6)	14.8 (12.1, 17.5)
	Bolivia	8.1 (5.4, 10.8)	68.9 (66.2, 71.6)	18.2 (15.3, 21.0)
	Guyana	8.1 (0.2, 16.0)	72.5 (64.9, 80.2)	24.5 (15.8, 33.2)
	Honduras	3.4 (1.7, 5.1)	66.7 (62.1, 71.3)	18.4 (15.7, 21.2)
UMIC	Namibia	3.4 (2.3, 4.5)	31.1 (28.4, 33.7)	8.0 (4.7, 11.4)
	Iraq	7.1 (4.0, 10.2)	65.6 (62.8, 68.4)	16.3 (12.5, 20.0)
	Malaysia	2.7 (1.7, 3.8)	54.3 (52.6, 56.1)	14.1 (12.1, 16.1)
	Tonga	7.0 (5.1, 8.9)	56.9 (55.1, 58.6)	18.6 (16.4, 20.7)
	Jamaica	6.2 (0.1, 12.4)	41.8 (38.6, 44.9)	16.4 (12.3, 20.6)
	Suriname	4.6 (3.6, 5.5)	62.2 (60.1, 64.2)	16.7 (13.5, 19.8)
HIC	Bahrain	5.7 (4.6, 6.8)	55.5 (53.2, 57.8)	24.2 (22.3, 26.1)
	Brunei	4.2 (2.7, 5.8)	54.7 (51.9, 57.5)	12.9 (10.1, 15.6)
	French Polynesia	7.8 (5.2, 10.3)	52.9 (51.2, 54.6)	18.9 (15.6, 22.2)
	Mauritius	2.7 (0.9, 4.4)	56.8 (55.0, 58.6)	26.9 (13.2, 40.5)
	Uruguay	1.3 (0.5, 2.0)	74.9 (73.2, 76.6)	40.2 (37.6, 42.7)

LIC-low income countries, LMIC-lower middle income countries, UMIC-upper middle income countries, HIC-high income countries, CI-confidence interval, AFR-African region, AMR-Region of the Americas, EMR-Eastern Mediterranean Region.

[a]Country income level was based on the World Bank classification at the year of the survey in the respective countries.

[b]"Went hungry most of the time or always because there was not enough food in their home during the past 30 days".

[c]"Ate fruits or vegetables five or more times per day during the past 30 days".

[d]"Were physically active for a total of at least 60 minutes per day on at least 5 days in the past 7 days".

[e]Prevalence were weighted, and adjusted for age and gender.

Supplementary Table S2. Country-wise estimates of the association between BMI categories and each independent variable (n=71,176), Global School-Based Student Health Survey, 2003-2017.

Income Level[a]	Country	Age[b] underweight AOR (95%CI)[h]	Age[b] overweight/obese AOR (95%CI)[h]	Gender[c] underweight AOR (95%CI)[h]	Gender[c] overweight/obese AOR (95%CI)[h]	Food insecurity[d] underweight AOR (95%CI)[h]	Food insecurity[d] overweight/obese AOR (95%CI)[h]
LIC	Benin	1.17 (0.84, 1.63)	0.67 (0.47, 0.97)	2.61 (1.57, 4.35)	0.63 (0.36, 1.09)	0.75 (0.37, 1.54)	1.33 (0.67, 2.62)
	Uganda	0.80 (0.63, 1.01)	0.93 (0.65, 1.33)	4.76 (3.08, 7.35)	0.15 (0.08, 0.30)	0.56 (0.28, 1.15)	1.02 (0.46, 2.29)
	Syria	0.90 (0.70, 1.17)	0.86 (0.75, 1.00)	1.32 (0.88, 2.00)	1.72 (1.34, 2.20)	1.40 (0.82, 2.40)	0.82 (0.54, 1.25)
	Subtotal	0.92 (0.75, 1.13)	0.84 (0.74, 0.96)	2.53 (1.17, 5.49)	0.56 (0.14, 2.24)	0.93 (0.64, 1.34)	0.95 (0.69, 1.32)
	I^2 (p value)	40.4% (p=0.187)	0.0% (p=0.385)	88.7% (p=0.000)	96.1% (p=0.000)	56.1% (p=0.102)	0.0% (p=0.487)
LMIC	Algeria	1.05 (0.92, 1.19)	0.91 (0.82, 1.02)	2.80 (2.19, 3.58)	0.59 (0.46, 0.75)	1.32 (0.83, 2.09)	0.76 (0.42, 1.39)
	China (Beijing)	1.15 (0.96, 1.39)	0.90 (0.75, 1.07)	1.50 (1.08, 2.09)	2.18 (1.53, 3.10)	0.90 (0.40, 2.00)	0.99 (0.46, 2.13)

	Pakistan	0.91 (0.80, 1.03)	1.03 (0.90, 1.17)	1.34 (0.84, 2.15)	0.58 (0.35, 0.96)	0.85 (0.60, 1.22)	1.08 (0.54, 2.15)
	Indonesia	1.16 (1.08, 1.25)	0.88 (0.80, 0.97)	2.16 (1.87, 2.50)	1.39 (1.12, 1.72)	0.76 (0.54, 1.07)	0.74 (0.49, 1.11)
	Mongolia	0.98 (0.85, 1.13)	0.90 (0.80, 1.01)	1.51 (1.08, 2.12)	1.24 (1.01, 1.52)	1.38 (0.50, 3.82)	1.12 (0.50, 2.51)
	Thailand	1.28 (1.18, 1.39)	0.81 (0.72, 0.91)	1.86 (1.48, 2.34)	2.22 (1.80, 2.75)	1.31 (0.88, 1.96)	0.92 (0.56, 1.51)
	Bolivia	1.15 (0.77, 1.71)	0.82 (0.70, 0.96)	1.60 (0.81, 3.18)	0.81 (0.68, 0.97)	0.62 (0.18, 2.11)	0.68 (0.42, 1.09)
	Guyana	1.11 (0.94, 1.31)	0.85 (0.68, 1.05)	1.67 (1.21, 2.31)	0.90 (0.64, 1.24)	0.69 (0.39, 1.22)	0.71 (0.33, 1.54)
	Honduras	1.13 (0.88, 1.44)	0.95 (0.82, 1.11)	2.14 (1.42, 3.23)	0.99 (0.78, 1.24)	1.01 (0.32, 3.19)	0.56 (0.21, 1.46)
	Subtotal	1.10 (1.01, 1.19)	0.89 (0.85, 0.94)	1.88 (1.61, 2.19)	1.09 (0.81, 1.48)	0.94 (0.80, 1.12)	0.81 (0.66, 0.98)
	I^2 (p value)	69.0% (p=0.001)	14.9% (p=0.310)	57.3% (p=0.016)	92.5% (p=0.000)	7.5% (p=0.373)	0.0% (p=0.920)
UMIC	Namibia	1.03 (0.87, 1.23)	1.12 (0.82, 1.53)	2.45 (1.80, 3.34)	0.60 (0.42, 0.85)	1.35 (0.87, 2.11)	0.89 (0.38, 2.06)

	Iraq	1.08 (0.84, 1.38)	0.80 (0.70, 0.92)	2.26 (1.25, 4.10)	0.87 (0.60, 1.28)	1.46 (0.67, 3.17)	0.83 (0.52, 1.34)
	Malaysia	1.02 (0.96, 1.09)	0.90 (0.85, 0.96)	1.89 (1.70, 2.11)	1.37 (1.26, 1.50)	1.18 (0.97, 1.44)	0.72 (0.59, 0.88)
	Tonga	0.92 (0.60, 1.42)	1.06 (0.96, 1.16)	3.07 (0.85, 11.05)	0.67 (0.54, 0.82)	1.91 (0.51, 7.12)	0.99 (0.72, 1.36)
	Jamaica	1.59 (1.12, 2.25)	0.93 (0.73, 1.18)	2.89 (1.32, 6.30)	0.88 (0.65, 1.19)	1.23 (0.45, 3.34)	1.07 (0.55, 2.11)
	Suriname	0.94 (0.77, 1.16)	0.96 (0.84, 1.09)	1.55 (1.14, 2.11)	1.02 (0.79, 1.30)	1.29 (0.73, 2.26)	0.92 (0.65, 1.30)
	Subtotal	1.04 (0.95, 1.14)	0.94 (0.86, 1.03)	1.97 (1.70, 2.28)	0.88 (0.64, 1.21)	1.23 (1.05, 1.45)	0.83 (0.72, 0.95)
	I^2 (p value)	30.6% (p=0.206)	66.0% (p=0.012)	19.1% (p=0.289)	91.7% (p=0.000)	0.0% (p=0.964)	0.0% (p=0.545)
HIC	Bahrain	1.02 (0.91, 1.14)	0.95 (0.89, 1.01)	2.29 (1.70, 3.08)	1.23 (1.07, 1.42)	1.22 (0.76, 1.97)	1.09 (0.91, 1.31)
	Brunei	1.18 (0.87, 1.62)	0.97 (0.87, 1.08)	1.88 (1.23, 2.87)	1.37 (1.06, 1.76)	1.15 (0.51, 2.62)	0.55 (0.33, 0.90)
	French Polynesia	0.77 (0.60, 0.99)	0.89 (0.78, 1.01)	1.02 (0.60, 1.70)	1.25 (0.99, 1.58)	0.22 (0.02, 2.00)	0.87 (0.63, 1.21)

	Mauritius	1.04 (0.90, 1.20)	0.85 (0.69, 1.04)	1.60 (1.20, 2.14)	1.56 (1.18, 2.07)	1.09 (0.67, 1.78)	0.92 (0.51, 1.63)
	Uruguay	1.13 (0.79, 1.61)	0.84 (0.73, 0.96)	1.44 (0.90, 2.32)	1.26 (1.05, 1.50)	1.64 (0.41, 6.63)	0.97 (0.34, 2.77)
	Subtotal	1.01 (0.90, 1.12)	0.93 (0.88, 0.97)	1.66 (1.30, 2.13)	1.29 (1.18, 1.41)	1.14 (0.84, 1.55)	0.90 (0.70, 1.14)
	I^2 (p value)	33.8% (p=0.196)	7.2% (p=0.366)	52.3% (p=0.079)	0.0% (p=0.637)	0.0% (p=0.675)	42.4% (p=0.139)
	Overall	1.05 (0.99, 1.11)	0.91 (0.88, 0.94)	1.92 (1.72, 2.15)	1.03 (0.88, 1.20)	1.08 (0.97, 1.20)	0.88 (0.81, 0.96)
	I^2 (p value)	61.5% (p=0.000)	35.7% (p=0.047)	61.1% (p=0.000)	90.3% (p=0.000)	0.8% (p=0.449)	0.0% (p=0.633)
Income Level[a]	Country	Tobacco use[e]		Fruit and vegetable consumption[f]		Physical activity[g]	
		underweight AOR (95%CI)[h]	overweight/obese AOR (95%CI)[h]	underweight AOR (95%CI)[h]	overweight/obese AOR (95%CI)[h]	underweight AOR (95%CI)[h]	overweight/obese AOR (95%CI)[h]
LIC	Benin	0.45 (0.08, 2.58)	0.53 (0.10, 2.73)	0.89 (0.39, 2.06)	0.89 (0.45, 1.75)	0.93 (0.50, 1.74)	1.35 (0.82, 2.21)
	Uganda	1.00 (0.51, 1.95)	2.30 (1.04, 5.09)	1.13 (0.81, 1.58)	1.11 (0.65, 1.91)	1.51 (1.15, 1.98)	1.21 (0.70, 2.09)
	Syria	0.98 (0.62, 1.56)	1.13 (0.78, 1.64)	1.48 (0.88, 2.50)	1.01 (0.78, 1.30)	0.76 (0.45, 1.28)	0.94 (0.64, 1.36)

	Subtotal	0.95 (0.66, 1.38)	1.30 (0.72, 2.37)	1.18 (0.91, 1.54)	1.01 (0.81, 1.26)	1.07 (0.67, 1.72)	1.10 (0.85, 1.44)
	I^2 (p value)	0.0% (p=0.687)	44.1% (p=0.167)	0.0% (p=0.541)	0.0% (p=0.882)	67.8% (p=0.045)	0.0% (p=0.487)
LMIC	Algeria	0.98 (0.62, 1.58))	1.71 (1.25, 2.34)	0.85 (0.54, 1.33)	1.77 (0.82, 3.81)	0.84 (0.58, 1.22)	0.69 (0.52, 0.91)
	China (Beijing)	0.66 (0.41, 1.06)	1.36 (0.97, 1.90)	1.23 (0.30, 5.00)	0.89 (0.38, 2.07)	1.35 (1.04, 1.76)	0.78 (0.59, 1.03)
	Pakistan	1.08 (0.71, 1.65)	1.24 (0.81, 1.88)	1.02 (0.70, 1.49)	0.99 (0.51, 1.93)	0.88 (0.61, 1.26)	0.73 (0.46, 1.17)
	Indonesia	0.76 (0.61, 0.94)	0.67 (0.50, 0.91)	0.91 (0.76, 1.08)	1.08 (0.85, 1.36)	0.75 (0.60, 0.94)	1.09 (0.91, 1.32)
	Mongolia	1.14 (0.69, 1.89)	1.22 (0.65, 2.26)	1.05 (0.74, 1.47)	1.17 (0.91, 1.49)	0.89 (0.65, 1.23)	0.63 (0.47, 0.83)
	Thailand	1.15 (0.80, 1.66)	1.06 (0.77, 1.44)	1.23 (0.90, 1.68)	0.81 (0.61, 1.07)	0.81 (0.62, 1.07)	0.78 (0.63, 0.96)
	Bolivia	1.00 (0.45, 2.22)	0.74 (0.52, 1.06)	0.95 (0.43, 2.08)	1.14 (0.84, 1.55)	1.26 (0.75, 2.10)	0.89 (0.68, 1.16)
	Guyana	0.97 (0.56, 1.68)	0.81 (0.54, 1.21)	1.24 (0.63, 2.42)	0.93 (0.57, 1.52)	0.54 (0.42, 0.71)	0.89 (0.61, 1.29)

	Honduras	0.47 (0.22, 1.03)	0.94 (0.57, 1.57)	0.76 (0.46, 1.28)	0.89 (0.63, 1.26)	0.81 (0.37, 1.78)	1.33 (0.91, 1.93)
	Subtotal	0.89 (0.76, 1.05)	1.03 (0.82, 1.30)	0.98 (0.87, 1.10)	1.03 (0.92, 1.16)	0.86 (0.70, 1.06)	0.85 (0.73, 0.98)
	I^2 (p value)	20.9% (p=0.257)	69.8% (p=0.001)	0.0% (p=0.777)	0.0% (p=0.505)	70.4% (p=0.001)	60.5% (p=0.009)
UMIC	Namibia	0.63 (0.38, 1.06)	1.45 (1.00, 2.12)	0.90 (0.68, 1.19)	1.65 (0.71, 3.82)	1.08 (0.83, 1.41)	1.30 (0.91, 1.87)
	Iraq	0.94 (0.49, 1.79)	0.96 (0.52, 1.80)	1.14 (0.58, 2.27)	0.64 (0.43, 0.95)	0.96 (0.52, 1.79)	1.12 (0.83, 1.52)
	Malaysia	1.05 (0.89, 1.22)	0.84 (0.73, 0.98)	0.82 (0.73, 0.93)	1.17 (1.02, 1.33)	0.83 (0.73, 0.93)	0.80 (0.73, 0.88)
	Tonga	1.24 (0.35, 4.45)	1.12 (0.86, 1.45)	3.07 (0.38, 24.83)	1.02 (0.82, 1.29)	0.81 (0.29, 2.29)	1.07 (0.87, 1.31)
	Jamaica	0.93 (0.38, 2.26)	0.73 (0.45, 1.23)	0.97 (0.45, 2.07)	1.24 (0.74, 2.09)	0.91 (0.53, 1.57)	1.01 (0.75, 1.37)
	Suriname	0.45 (0.24, 0.84)	1.04 (0.67, 1.61)	0.80 (0.41, 1.57)	1.64 (0.92, 2.92)	0.75 (0.44, 1.28)	0.88 (0.64, 1.21)

	Subtotal	0.82 (0.60, 1.12)	1.00 (0.82, 1.22)	0.84 (0.76, 0.94)	1.11 (1.00, 1.23)	0.87 (0.78, 0.96)	0.99 (0.84, 1.17)
	I^2 (p value)	48.0% (p=0.087)	51.3% (p=0.068)	0.0% (p=0.733)	55.9% (p=0.045)	0.0% (p=0.612)	67.2% (p=0.009)
HIC	Bahrain	0.82 (0.59, 1.15)	0.86 (0.72, 1.03)	1.06 (0.78, 1.44)	1.08 (0.94, 1.23)	1.13 (0.89, 1.42)	0.72 (0.64, 0.81)
	Brunei	0.43 (0.15, 1.25)	0.86 (0.58, 1.26)	0.62 (0.39, 1.00)	1.21 (0.91, 1.60)	0.58 (0.32, 1.04)	0.58 (0.42, 0.79)
	French Polynesia	0.28 (0.15, 0.56)	1.30 (0.94, 1.80)	1.01 (0.39, 2.65)	0.83 (0.66, 1.05)	0.62 (0.31, 1.22)	0.78 (0.64, 0.96)
	Mauritius	1.17 (0.74, 1.86)	0.81 (0.59, 1.12)	0.91 (0.66, 1.27)	1.09 (0.73, 1.63)	0.90 (0.61, 1.32)	0.87 (0.63, 1.21)
	Uruguay	0.68 (0.29, 1.61)	1.13 (0.80, 1.59)	1.14 (0.60, 2.19)	1.00 (0.83, 1.20)	0.55 (0.25, 1.19)	0.81 (0.65, 1.01)
	Subtotal	0.65 (0.39, 1.06)	0.96 (0.81, 1.14)	0.93 (0.77, 1.12)	1.03 (0.94, 1.13)	0.81 (0.60, 1.10)	0.74 (0.68, 0.82)
	I^2 (p value)	70.5% (p=0.009)	42.1% (p=0.141)	0.0% (p=0.410)	23.5% (p=0.265)	51.5% (p=0.083)	7.9% (p=0.362)

Overall	0.85 (0.74, 0.97)	1.02 (0.91, 1.14)	0.92 (0.86, 0.99)	1.05 (0.99, 1.11)	0.89 (0.79, 1.00)	0.87 (0.80, 0.95)
I^2 (p value)	41.3% (p=0.021)	56.7% (p=0.000)	0.0% (p=0.599)	13.9% (p=0.271)	60.3% (p=0.000)	60.4% (p=0.000)

LIC-low income countries, LMIC-lower middle income countries, UMIC-upper middle income countries, HIC-high income countries, CI-confidence interval, AFR-African region, AMR-Region of the Americas, EMR-Eastern Mediterranean Region.

[a]Country income level was based on the World Bank classification at the year of the survey in the respective countries.

[b]Age was analyzed as a continuous variable, with 12 as the reference category.

[c]Female was the reference category.

[d]"Never, rarely, sometimes went hungry because there was not enough food in their home during the past 30 days" was the reference category.

[e]Never used any tobacco products in the past 30 days was the reference category.

[f]"Ate fruits or vegetables ≤4 times per day during the past 30 days".

[g]"Were physically active for a total of at least 60 minutes per day for ≤4 days in the past 7 days".

© 2021 Wang Q.
